# Histone deacetylases: potential therapeutic targets in cisplatin-induced acute kidney injury

**DOI:** 10.1080/07853890.2024.2418958

**Published:** 2024-10-25

**Authors:** Shuxian Guo, Jin Zhao, Yuzhan Zhang, Yunlong Qin, Jinguo Yuan, Zixian Yu, Yan Xing, Yumeng Zhang, Yueqing Hui, Anjing Wang, Mei Han, Yueru Zhao, Xiaoxuan Ning, Shiren Sun

**Affiliations:** a Department of Nephrology, Xijing Hospital, Air Force Medical University, Xi’an, China; b School of Clinical Medicine, Health Science Center, Xi’an Jiaotong University, Xi’an, China; c Department of Geriatric, Xijing Hospital, Air Force Medical University, Xi’an, China

**Keywords:** Histone deacetylase, nephrotoxicity, cisplatin, onconpehrology

## Abstract

**Aim:** Chemotherapy has been well shown to enhance life expectancy in patients with malignancy. However, conventional chemotherapy drugs, particularly cisplatin, are highly associated with nephrotoxicity, which limits therapeutic efficacy and impairs quality of life. Histone deacetylases (HDACs) are proteases that play significant roles in diseases by influencing protein post-translational modification and gene expression. Agents that inhibit HDAC enzymes have been developed and approved by the FDA as anticancer drugs. It is worth noting that in certain preclinical studies with tumour cell lines, the integration of HDAC modulators and cisplatin not only exerts synergistic or additive tumour-killing effects but also alleviates cisplatin nephrotoxicity. The aim of this review is to discuss the role of HDACs in cisplatin nephrotoxicity.

**Methods:** After searching in PubMed and Web of Science databases using ‘Histone deacetylase’, ‘nephrotoxicity’, ‘cisplatin’, and ‘onconpehrology’ as keywords, studies related was compiled and examined.

**Results:** HDAC inhibitors exert renal protective effects by inhibiting inflammation, apoptosis, oxidative stress, and promoting autophagy; whereas sirtuins play a renal protective role by regulating lipid metabolism, inhibiting inflammation and apoptosis, and protecting mitochondrial biosynthesis and mitochondrial dynamics. These potential interactions provide clues concerning targets for molecular treatment.

**Conclusion:** This review encapsulates the function and molecular mechanisms of HDACs in cisplatin nephrotoxicity, providing the current view by which HDACs induce different biological signaling in the regulation of chemotherapy-associated renal injury. More importantly, this review exhaustively elucidates that HDACs could be targeted to develop a new therapeutic strategy in treating cisplatin nephrotoxicity, which will extend the knowledge of the biological impact and clinical implications of HDACs.

## Introduction

Cancer stands as a prominent contributor to mortality and morbidity worldwide. The past decade has witnessed tremendous progress in novel approaches to treat cancer, but traditional antineoplastic agents, such as cisplatin, remain the mainstay of therapeutic options for solid tumors [1]. It is estimated that 50% of patients receive cisplatin as part of their chemotherapy regimen [2]. However, approximately 30% of patients treated with cisplatin occur kidney injury, which increases mortality and morbidity in patients with cancer. As a result, the utility of cisplatin chemotherapy is restricted [3,4].

At present, several kinds of countermeasures are used to treat the renal damage caused by cisplatin, but a majority of measures show varying degrees of deficiency. For instance, forced diuresis induced by hydration, mannitol, or magnesium supplementation attenuates or prevents cisplatin nephrotoxicity in clinical treatment [5]. However, given the large volume of liquid required and the slow onset of effect, these conditions may lead to over-diuresis, dehydration, and an increase of the cardiac load, which considerably hampers their utilization in high-dose cisplatin-treated patients [6]. Therefore, novel approaches targeting cisplatin-induced nephrotoxicity pathways may achieve better protective effects on the kidneys.

Histone deacetylases (HDACs) are widely recognized as a family of proteases that catalyze histone deacetylation within the nucleus, which are involved in the modification of chromosomal structure and regulation of gene expression [7]. The superfamily of HDACs in mammals comprises 18 isoenzymes, which can be segmented into four categories based on their homology with yeast proteins: class I HDAC (HDAC1, 2, 3, 8), class II HDAC (class IIa, HDAC4, 5, 7, and 9; class IIb 6 and 10), class III HDAC (sirtuins, SIRT1-7) and class IV HDAC (HDAC11) [8]. Previous studies have indicated that HDACs are a promising chemotherapy target, with certain HDAC inhibitors already approved by the FDA as anticancer agents [9]. Notably, combining HDACs and cisplatin in preclinical studies has shown synergistic or additive tumor killing effects while protecting kidneys from cisplatin nephrotoxicity [10,11]. HDACs, as well as their broad-spectrum or specific regulators, are implicated in multiple pathophysiological processes of cisplatin-induced renal injury, such as mitochondrial damage, oxidative stress, apoptosis, autophagy, and inflammatory response [3,4]. Consequently, compounds modifying HDACs may be a promising treatment for cisplatin-induced nephrotoxicity and/or as adjuncts to cisplatin in the treatment of cancer [12,13]. In this paper, we present a comprehensive overview of the pivotal role of HDACs in the pathogenesis of cisplatin nephrotoxicity and summarize the clinical translation and therapeutic implications of HDAC inhibitors (HDACi) and SIRT activators.

## Expression and function of HDACs in the pathogenesis of cisplatin nephrotoxicity

**Figure 1.**
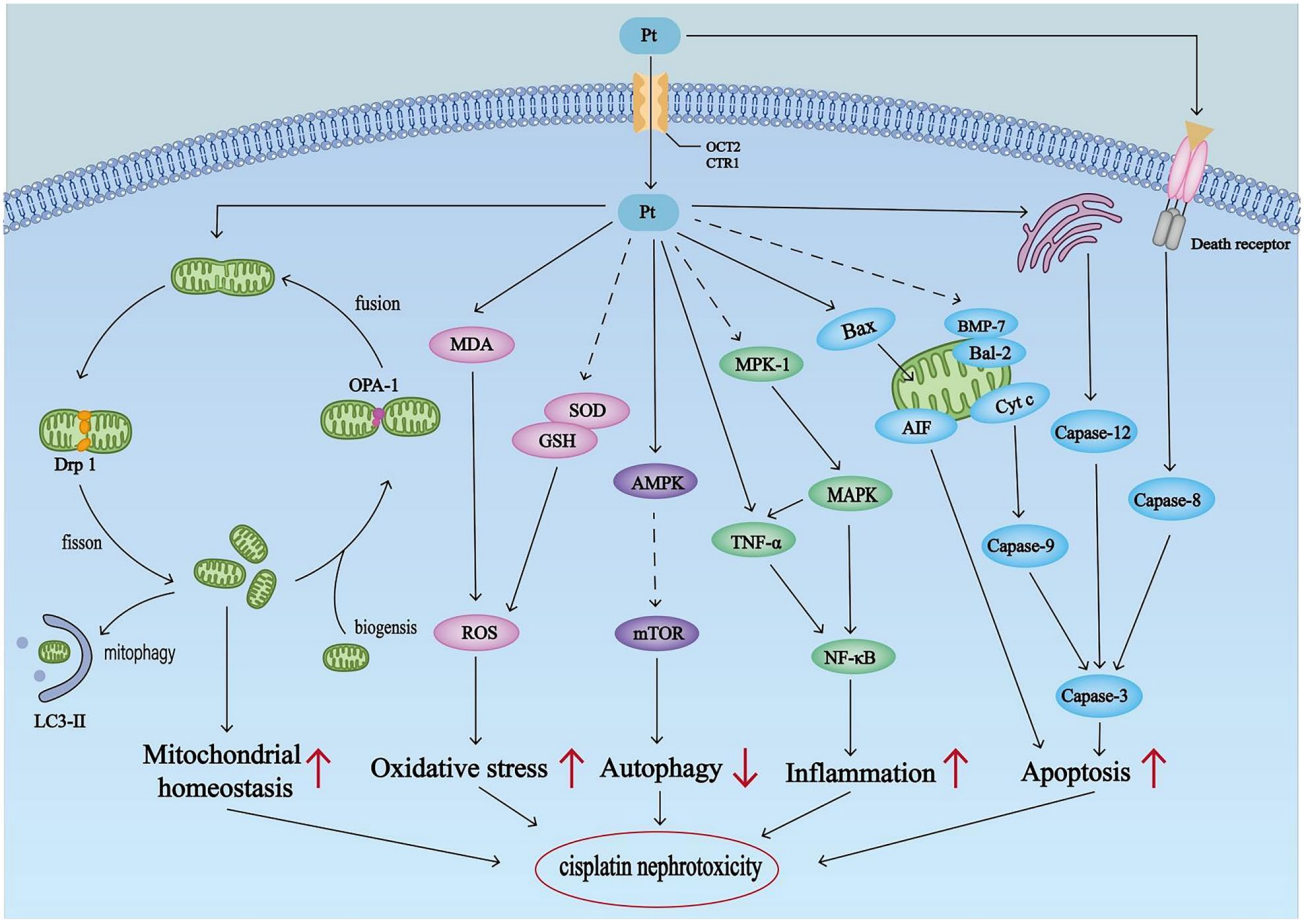
Molecular mechanisms of cisplatin nephrotoxicity. OPA1: optic atrophy 1; DRP1: dynamin-related protein 1; MDA: malonaldehyde; GSH: glutathione; SOD: superoxide dismutase; ROS: reactive oxygen species; AMPK: AMP‑activated protein kinase; mTOR: mechanistic target of rapamycin; MAPK: mitogen-activated protein kinase; TNF-α: tumour necrosis factor-α; NF-κB: factor-kappaB; Bax, BCL-2-associated X protein; Bcl-2, B-cell lymphoma 2; BMP-7: morphogenetic protein 7; Cyt C: cytochrome c; AIF: apoptosis-inducing factor.

The pathogenesis of cisplatin-induced nephrotoxicity encompasses a spectrum of intricate processes (Figure 1). In the kidneys, renal tubular epithelial cells (RTECs) are the prominent targets of cisplatin [14]. Key transporters, such as organic cation transporter 2 (OCT2) and copper transporter 1 (CTR1), are accountable for the uptake of cisplatin from the blood into RTECs, resulting in greater cisplatin concentrations than in the blood [15,16]. Massive cisplatin could fleetly accumulate in the mitochondria and destroy mitochondrial homeostasis, indicating that mitochondria are the major organelle that receives cisplatin [17,18]. Mitochondrial dysfunction often gives rise to an augmented level of endogenous reactive oxygen species (ROS). The excessive ROS, in turn, affects the function of mitochondrial complex enzymes I–IV and impedes the smooth progression of the oxidative respiratory chain, ultimately forming a vicious cycle, causing cellular damage, and triggering inflammatory responses [19]. Meanwhile, cisplatin could quickly react with sulfur-containing antioxidants such as glutathione (GSH) and superoxide dismutase (SOD), causing the inactivation of antioxidants, the generation of lipid peroxidation products, and the production of ROS, all of which contribute to elevated oxidative stress and kidney injury [20]. Cisplatin can also activate apoptotic pathways within renal cells to aggravate kidney damage. Research has shown that cisplatin could elicit the extrinsic apoptosis pathway through the interaction between death receptors and intrinsic caspases, as well as affect the intrinsic apoptotic pathways via the endoplasmic reticulum stress (ERS) or mitochondrial dysfunction [21–23]. Furthermore, cisplatin increases the secretion of multiple proinflammatory cytokines including tumour necrosis factor-alpha (TNF-α) and interleukin-6 (IL-6), consequently triggering the activation of nuclear factor-kappa B (NF-κB), which exacerbates the renal damage and ultimately results in an unfavourable prognosis [24,25]. Although the presence of oxidative stress, apoptosis, and inflammatory response is detrimental in cisplatin-induced AKI, autophagy seems to exert a protective effect on RTECs. By activating the 5′-AMP-activated protein kinase (AMPK), a pivotal promoter of autophagy, cisplatin sets in motion a chain reaction that results in the inhibition of the mechanistic target of rapamycin complex 1 (mTORC1), ultimately triggering the autophagy and effectively shielding against kidney injury [26,27].

**Figure 2.**
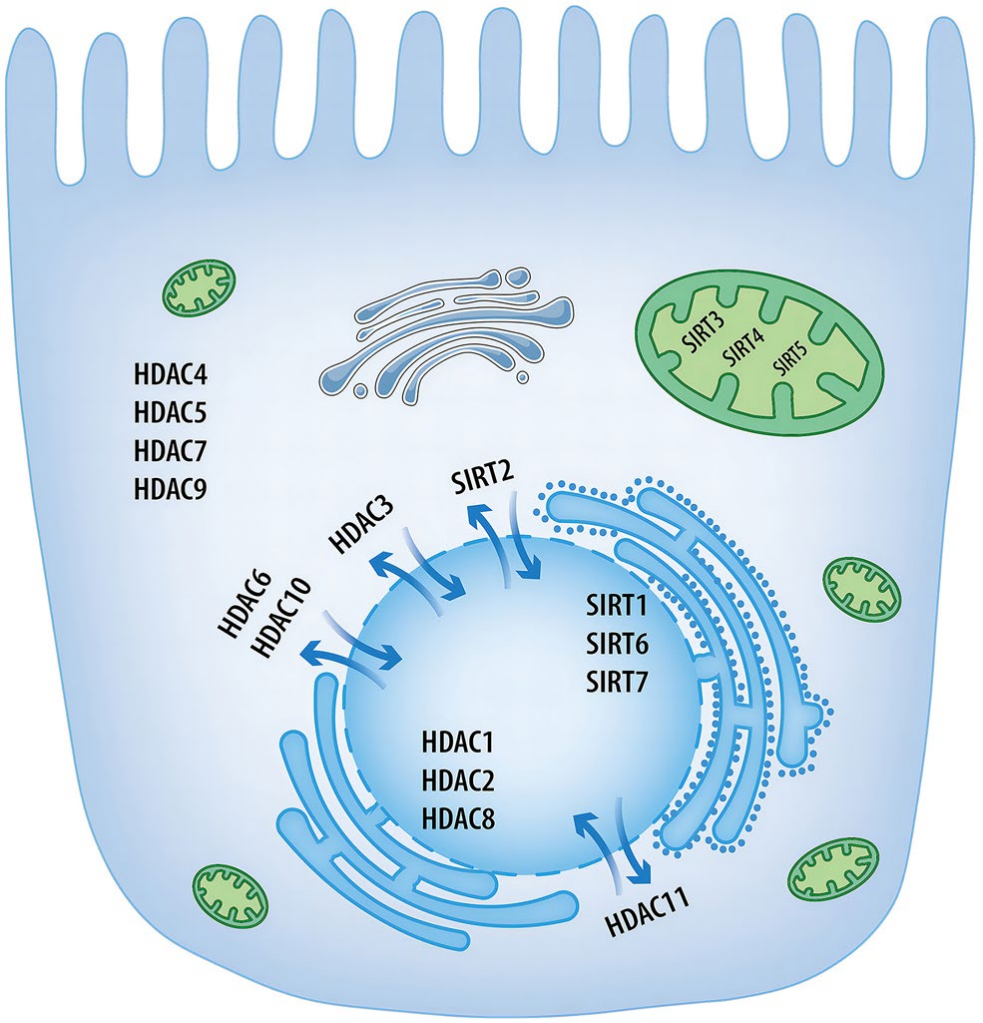
The distribution of histone deacetylases in proximal tubular epithelial cells. HDACs are divided into four classes based on their homology with yeast proteins. Except for HDAC3 in the nucleus and cytoplasm, class I HDACs are predominantly expressed in the nucleus. Class IIa HDACs are mainly located in the cytoplasm, while class IIb and IV HDACs can translocate between the nucleus and cytosol. As for sirtuins, SIRTl, SIRT6 and SIRT7 are distributed in the nucleus, SIRT2 shuttles between the nucleus and the cytoplasm, and SIRT3∼SIRT5 are distributed in the mitochondria. The localization of HDACs in the cells is dynamically changed. HDAC: histone deacetylase; SIRT: sirtuin.

These mechanisms underlying cisplatin-induced nephrotoxicity shed light on their potential as therapeutic avenues for ameliorating renal injury. HDACs are involved in the cisplatin-induced nephrotoxicity via influencing aforesaid pathogenesis. Class I, II and IV HDACs are Zn^2+^-dependent proteases, whereas sirtuins are nicotinamide adenine dinucleotide (NAD^+^)-dependent proteases. These deacetylases vary in deacetylase activity and cellular distribution (Figure 2). It was found that the inhibition of class I, II, and IV HDACs while the activation of the majority of sirtuins exerts a nephroprotective function in the pathogenesis of cisplatin kidney injury. Here we describe the effect of Zinc-dependent proteases (class I, II and IV HDACs) and sirtuins separately.

## Potential roles of zinc-dependent HDAC family in cisplatin nephrotoxicity

### HDAC2 promotes apoptosis

HDAC2, as a member of the class I HDAC family, is implicated in cellular homeostasis and organ development by modulating gene expressions [28]. Notably, a reduction in HDAC2 expression levels has been observed to coincide with elevated levels of acetylated histone H3 and involved in the renoprotective effects against kidney injury [29]. In cisplatin-induced AKI models, HDAC2 binds to the promoter region of bone morphogenetic protein 7 (BMP-7), a neotype antiapoptotic protein, thereby significantly downregulating its activity, and promoting RTECs apoptosis [30]. Consequently, the downregulation of HDAC2 or its downstream pathways may effectively impede apoptosis induced by cisplatin and offer promising therapeutic prospects for managing cisplatin-induced nephrotoxicity.

### HDAC5 favors anti-apoptosis

HDAC5 belongs to the class IIa HDAC subunit and has been observed to be upregulated in both AKI patients and animals [31]. It has been uncovered that HDAC5 attenuates the cisplatin-mediated activation of certain caspases, including caspase-3 and caspase-9, partially through an intrinsic apoptotic pathway associated with DNA damage [32]. Furthermore, when the HDAC5 gene is silenced, the suppression of cisplatin-induced cleavage of caspase-3 is canceled [32]. Collectively, these outcomes substantiate that HDAC5 diminishes the cisplatin-triggered apoptosis of RTECs. Further investigations are warranted to unravel the intricate correlation between HDAC5 and cisplatin-induced nephrotoxicity in RTECs, as HDAC5 exhibits divergent apoptotic effects in contrast to other Zinc-dependent HDACs.

### HDAC6 facilitates nephrotoxicity

HDAC6, classified within the class IIb HDAC subfamily, encompasses a conserved C-terminal ubiquitin-binding domain and two N-terminal deacetylating domains and mainly localizes in the cytoplasm [33]. It governs a multitude of crucial biological processes and displays distinctive substrate specificity towards non-histone proteins [34]. Notably, cisplatin-induced nephrotoxicity coincided with heightened expression and activation of HDAC6 [35]. E-cadherin is a transmembrane molecule that plays a vital role in facilitating cell-cell adhesion among epithelial cells [36]. The loss of E-cadherin disrupts cell adhesion, leading to cellular detachment from the extracellular matrix and triggering cell apoptosis [36]. Research indicates that cisplatin treatment reduced the expression of E-cadherin in cultured human kidney 2 (HK2) cells while inhibition of HDAC6 partially preserved E-cadherin expression [37]. Therefore, by inhibiting HDAC6 and preserving the expression of E-cadherin, it is possible to enhance the survival of RETCs. Likewise, an additional investigation revealed that inhibition of HDAC6 remarkably hindered ERS, substantiated by the notable decline in protein expression of the glucose-regulated protein (GRP78) and X-box binding protein 1 (XBP1) [38]. Down-regulated GRP78 reduces the release of double-stranded RNA-activated protein kinase-like ER kinase (PERK), which limits eIF2α phosphorylation, regulating tubular epithelial cell apoptosis [39]. In addition, blocking HDAC6 also inhibits oxidative stress, autophagy, and inflammation. This renoprotective function of HDAC6 blockade is related to the activation of SOD, enhancement of Beclin-1 and autophagy-related protein 7 (Atg7), as well as suppression of NF-κB signaling [37]. Notwithstanding HDAC6 shows promise as a potential therapeutic target for cisplatin-induced nephrotoxicity, further research is needed to fully elucidate its precise mechanisms and evaluate the safety and efficacy of HDAC6 inhibitors.

### Other zinc-dependent HDACs may likewise exert a significant role

Remarkably, considerable studies have also documented the crucial role of other HDACs in non-cisplatin-induced AKI models, implying that they might affect analogous pathways in cisplatin-induced AKI. For instance, HDAC1 can bind to the inflammatory cytokine gene containing the NF-κB binding sites, which suppresses inflammatory cytokine transcription and mitigates the inflammatory response through chromatin structure remodeling [40]. HDAC4 may promote renal repair after AKI by participating in the proliferative response of renal tubules [41]. HDAC8 is capable of reducing the level of histone H3 acetylation on the dynamin-related protein 1 (DRP1) promoter, and suppressing DRP1 expression and mitochondrial fission, which reduces mitochondrial dysfunction and histiocytic damage [42,43]. HDAC11 has been shown to inhibit histone H3 acetylation or plasminogen activator inhibitor type-1(PAI-1) expression, dampening oxidative stress and inflammatory responses [44]. Additionally, in renal carcinoma cells, HDAC7 and HDAC9 impair the enrichment of H3K18ac and H3K27ac modifications in the OCT2 promoter, leading to the transcriptional repression of OCT2 [45,46]. This signifies that HDAC7 and HDAC9 can exert nephroprotective effects by reducing cisplatin uptake through regulating the expression and activity of OCT2. Overall, these findings further emphasize the potential importance of HDACs in the pathogenesis and prevention of kidney injury. While the role of these HDACs will need to be further verified in a model of cisplatin-induced nephrotoxicity.

## Potential roles of NAD^+^-dependent HDAC family in cisplatin nephrotoxicity

### SIRT1 mitigates nephrotoxicity

SIRT1, the most extensively studied sirtuins, intricately participates in the regulation of manifold physiological functions. Not only can they deacetylate histones, but they also act on significant momentous transcriptional regulators such as p53, peroxisome proliferator-activated receptor gamma (PPARγ) coactivator 1-alpha (PGC-1α), and NF-κB, regulating fatty acid metabolism, apoptosis, and inflammation processes [47]. Hasegawa et al. [48] first identified the function of SIRT1 in the kidney. On the one hand, after cisplatin treatment, the number and function of peroxisomes in kidney tissues were suppressed, leading to the inhibition of FAO activity and a decrease in catalase expression. Nevertheless, SIRT1 overexpression in RTECs can restore the amount and function of peroxisomes, thereby facilitating the elimination of ROS and the recovery from mitochondrial dysfunction [49]. On the other hand, upregulation of SIRT1 by a specific agonist can effectively reduce macrophage infiltration, alleviate the oxidation response, and inhibit fatty acid deposition in RTECs [49]. p53 is confirmed as a downstream substrate of SIRT1 [50]. Upon binding with SIRT1, the C-terminal lysine residue of p53 is deacetylated, resulting in its subsequent inactivation [51]. Extensive cisplatin-induced AKI animal research has emphasized the crucial role of the SIRT1/p53 signaling pathway in apoptosis regulation [52]. SIRT1 suppresses the acetylation of p53 and consequently inhibits the apoptotic cascade by downregulation of PUMA-α and Bax, as well as activation of caspase-3, which ultimately ameliorates cisplatin-induced nephrotoxicity [52]. In addition, SIRT1 was dramatically down-regulated following repeated low-dose cisplatin treatment, resulting in increased acetylation of p65 and subsequent NF-κB activation, which increases the expression of various pro-inflammatory cytokines in tubule cells and induces maladaptive kidney repair [53,54].

### SIRT2 promotes inflammation and apoptosis

SIRT2, the inaugural sirtuin to be discovered, is essential for renal proinflammatory response and apoptosis [55]. A recent inquiry has brought to light the intricate connection between the proinflammatory effects of SIRT2 and mitogen-activated protein kinase phosphatase-1 (MKP-1). The absence of SIRT2 will increase the acetylation of MKP-1 and reverse the suppressed expression of MKP-1 caused by cisplatin treatment, which specifically impedes the phosphorylation of p38 and c-Jun, thus diminishing inflammation and apoptosis, ultimately ameliorating cisplatin-induced nephrotoxicity [56].

### SIRT3 maintains mitochondrial homeostasis

Located primarily in the mitochondrial matrix, SIRT3 has a global lysine deacetylase activity in mitochondria and is pivotal to maintaining mitochondrial homeostasis [57,58]. Prior investigations have evidenced that SIRT3 can attenuate the binding affinity of cisplatin to mitochondrial DNA, thereby preserving mitochondrial homeostasis and mitigating kidney injury [59]. According to research findings, the suppression of SIRT3 prompted the recruitment of mitochondrial fission factor (MFF) and DRP1 to the outer mitochondrial membrane, ultimately leading to mitochondrial fission and fragmentation [60]. Moreover, other studies also pointed out that SIRT3 alleviated cisplatin-induced kidney injury by maintaining mitochondrial integrity and reducing mitochondrial division and membrane depolarization [61–63]. Apart from regulating mitochondrial homeostasis, SIRT3 is also involved in the deacetylation of mitochondrial proteins to regulate several cellular pathways, such as oxidative stress, inflammatory responses, apoptosis, and energy metabolism [64]. Sang et al. [65] discovered that excessive activation of SIRT3 reduces oxidative stress in RTECs by modifying antioxidant defense systems, and ultimately alleviates nephrotoxicity caused by cisplatin. Kim et al. [66] observed that SIRT3 reduces the inflammatory cell infiltration and monocyte chemoattractant protein-1(MCP-1) expression, thereby attenuating cisplatin-induced kidney damage via inhibiting apoptosis and inflammatory reaction. Besides, the activation of SIRT3 also mitigates systemic inflammation by inhibiting the nuclear translocation of NF-κB [67]. Deficiency of SIRT3 reportedly aggravates lipid deposition and FAO dysfunction in cisplatin-mediated AKI, whereas the activation of SIRT3 improves the level of FAO by deacetylation of liver kinase B1 (LKB1) and activation of AMPK, which meets energy requirement and reduces the kidney damage [68]. Interestingly, SIRT3 has also been reported to antagonize cisplatin-induced ototoxicity through modulation of GLUT4 translocation and rescue of impaired glucose uptake caused by cisplatin [69]. This not only reveals a more extensive role for SIRT3 in alleviating cisplatin toxicity, but also provides new perspectives for the study of cisplatin-induced nephrotoxicity.

### SIRT4 may curtail inflammatory pathways

SIRT4, situated within the mitochondrial matrix, plays a critical role in energy metabolism [70]. Due to its ability to constrain glutamine dehydrogenase through ADP-ribosylation, SIRT4 is considered a ‘glutamine gatekeeper’ [71]. Recent emerging evidence indicates a remarkable decrease in SIRT4 levels following cisplatin administration, thus highlighting its involvement in the pathogenesis of cisplatin nephrotoxicity [72]. SIRT4 has been found to potentially form a complex with forkhead box M1 (FOXM1), which inhibits NF-κB signaling and the NLRP3 inflammasome, thereby alleviating inflammatory response and kidney injury [73]. The findings suggest that SIRT4 holds promise as a potential target for mitigating renal injury caused by cisplatin, despite the exact mechanism remains unclear.

### SIRT5 regulates oxidative stress and fatty acid metabolism

SIRT5, as the exclusive sirtuin that prefers malonyllysine, glutaryllysine, and succinyllysine as substrates, primarily resides in mitochondria and is involved in various biological processes during cisplatin treatment, such as reactive oxygen defense, apoptosis, and fatty acid metabolism [74,75]. The level of SIRT5 was decreased in cisplatin-treated renal tubular cells. However, the restoration of SIRT5 can reduce the accumulation of ROS, which maintains mitochondrial membrane potential and structural integrity, thereby alleviating mitochondrial damage [76]. Moreover, SIRT5 upregulates nuclear factor red lineage 2-related factor 2 (Nrf2), hemeoxygenase-1(HO-1), and B-cell lymphoma 2 (Bcl-2) expression, which reduces cytochrome c release and inhibits apoptosis cascade, inhibiting apoptosis progress [76]. Contrarily, another study demonstrated that SIRT5 exacerbated cisplatin-induced AKI. SIRT5 deletion disrupted the balance of FAO between mitochondria and peroxisomes by hypersuccinylation, blocking mitochondrial FAO while compensating for peroxisomal FAO [77]. This metabolic alteration reduces oxidative stress and fulfills the energy demands, ultimately protecting the kidneys from injury [77]. These contrasting findings indicate that the role of SIRT5 in cisplatin-induced nephrotoxicity is complex and may depend on various factors, including specific cell types and experimental conditions. Additional investigation is imperative to fully clarify the exact mechanisms underlying the involvement of SIRT5 in cisplatin-induced nephrotoxicity.

### SIRT6 inhibits inflammation, apoptosis, and oxidative stress

SIRT6, predominantly localizes within the nucleus, playing a significant role in AKI *via* modulating oxidative stress, apoptosis, and inflammation [78]. Extracellular signal-regulated kinases (ERK1/2) are proapoptotic signaling moieties that undergo activation upon DNA damage [79]. The activation of ERK1/2 is pivotal for the phosphorylation of p65 and subsequent activation of NF-κB [80]. Within renal tissues, SIRT6 inhibited ERK1/2 by means of histone H3K9 deacetylation, which suppresses NF-κB and p53 signaling, leading to the attenuation of cisplatin-induced inflammation and apoptosis [81]. Nrf2 is implicated in the regulation of oxidative stress and subsequent inflammation, thereby inhibiting the progression of renal injury [82]. A substantial body of studies has shown that SIRT6 exhibits antioxidative properties by enhancing the pharmacological efficacy of Nrf2 [83,84]. The cumulative findings from these studies strongly indicate that SIRT6 could potentially serve as a novel target for mitigating kidney injury.

### SIRT7 triggers inflammatory reactions

SIRT7, mainly involved in H3K18 deacetylation, has gradually drawn attention in regulating renal injury and has been shown to participate in renal diseases by triggering inflammation and apoptosis [85]. Multiple researches have proved that SIRT7 deficiency can provide renoprotection by reducing the secretion of various pro‐inflammatory cytokines and chemokines, as well as inhibiting inflammatory cell infiltration in the kidney [86]. Additionally, the absence of SIRT7 can limit the nuclear translocation of phosphor-p65, a subunit of NF-κB, thereby impeding the pro-inflammatory activity of NF-κB and safeguarding renal functionality [87,88].

**Figure 3.**
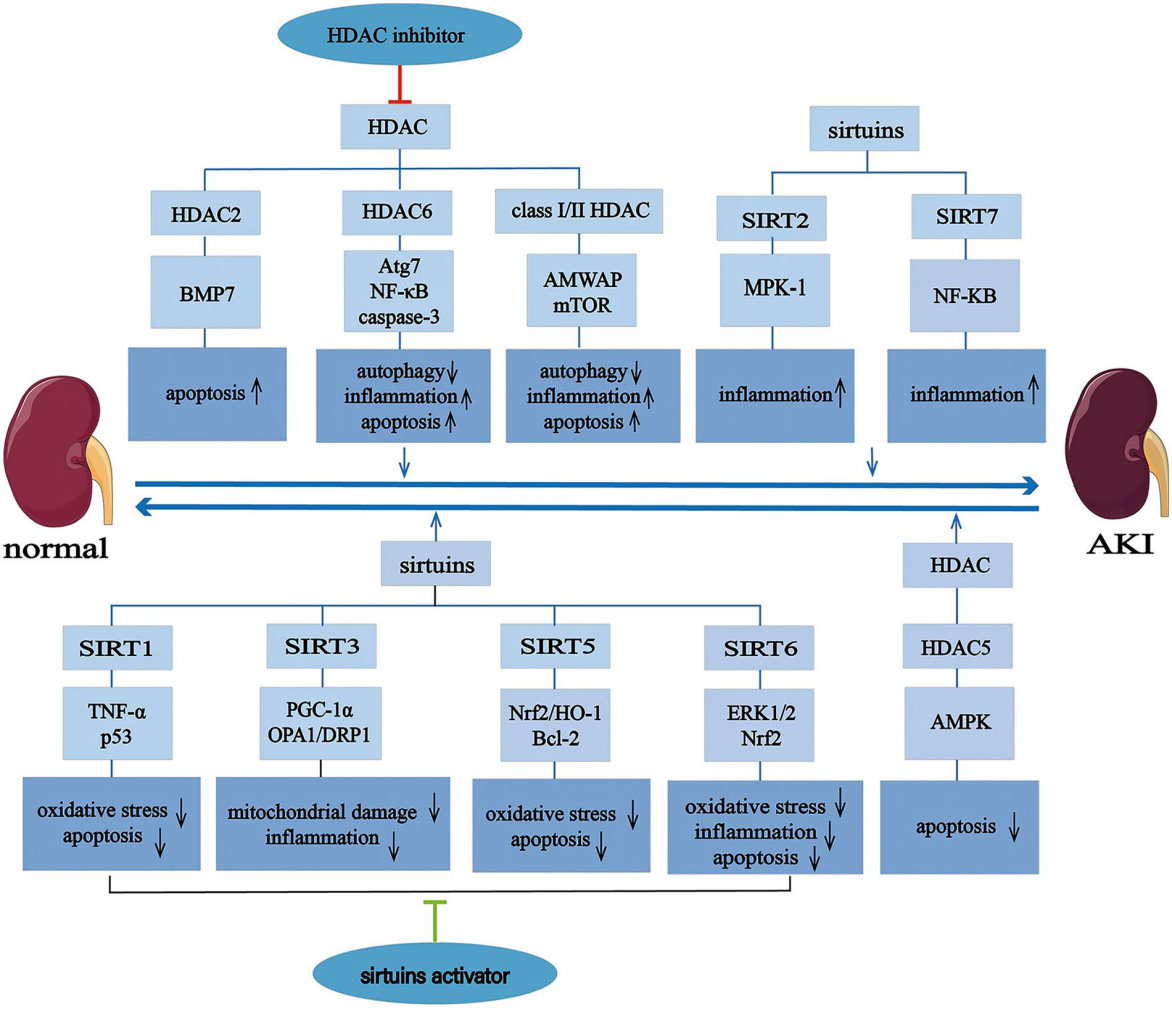
Possible renoprotective benefits of HDACs in cisplatin nephrotoxicity. HDAC inhibitors exert renal protective effects by inhibiting inflammation, apoptosis, oxidative stress, and promoting autophagy; whereas sirtuins play a renal protective role by regulating lipid metabolism, inhibiting inflammation and apoptosis, and protecting mitochondrial biosynthesis and mitochondrial dynamics. BMP-7: morphogenetic protein 7; AMPK: AMP‑activated protein kinase; Atg7: autophagy related protein 7; NF-κB: factor-kappaB; AMWAP: macrophage WAP domain protein; mTOR: mechanistic target of rapamycin; TNF-α: tumour necrosis factor-α;OPA1: optic atrophy 1; PGC-1α: PPARγ coactivator 1; DRP1: dynamin related protein 1; Nrf2: nuclear factor red lineage 2-related factor 2; Bcl-2, B-cell lymphoma 2; HO-1: heme oxygenase-1; MKP-1: mitogen-activated protein kinase phosphatase-1.

This section summarizes the expression and function of different deacetylases in the pathogenesis of cisplatin nephrotoxicity. In the zinc-dependent HDAC family, HDAC2 and HDAC6 promote apoptosis onset and aggravate renal injury, whereas HDAC5 exerts a renoprotective effect by inhibiting apoptosis. In the NAD^+^-dependent HDAC family, the positive effects of SIRT1, SIRT3, SIRT5, and SIRT6 in cisplatin kidney injury have been observed. Research has shown that SIRT1, SIRT5, and SIRT6 exert renal protective effects by regulating lipid metabolism and inhibiting inflammation and apoptosis. SIRT3 plays a renal protective role by protecting mitochondrial biosynthesis and mitochondrial dynamics. Of note, SIRT2 and SIRT7 might aggravate the development of AKI by promoting inflammation (Figure 3). Nevertheless, as cisplatin has multiple targets in the cells, blocking a single injurious event may only provide limited safeguarding for renal function. Regardless of whether inflammatory, oxidative stress or apoptosis pathways are blocked, protective effects can vary from marginal to impressive levels, but rarely complete. Therefore, the amalgamation of several drugs to achieve clinically meaningful results is possible and necessary. Furthermore, when considering combination strategies, the best choice is to utilize inhibitors or agonists targeting different pathways (Table 1).

**Table 1.** Possible renoprotective role of HDACs in cisplatin-induced nephrotoxicity classified by mechanisms.

Mechanisms	HDACs	Target and effects	Refs.
Energy metabolism	SIRT1	Restores the amount and function of peroxisomes	[48, 49]
	SIRT3	Improves the level of FAO by deacetylation of LKB1 and activation of AMPK	[68]
	SIRT5	Disruptes the balance of FAO between mitochondria and peroxisome by hypersuccinylation	[77]
Mitochondrial homeostasis	SIRT3	Decreases the recruitment of DRP1 and MFF to the outer mitochondrial membrane	[60]
	SIRT5	Maintains mitochondrial membrane potential and structural integrity	[76]
Oxidative stress	HDAC6	Reduces SOD activity	[37]
	SIRT1	Alleviates the oxidation response after cisplatin induction	[49]
	SIRT3	Modifies antioxidant defense systems	[65]
	SIRT5	Reduces ROS	[76]
	SIRT6	Improves the pharmacological activity of Nrf2	[83, 84]
Autophagy	HDAC6	Decreases the expression of Atg7 as well as Beclin-1, two conjugation system members in autophagosome formation	[37]
	SIRT6	Increases LC3-II and autophagolysosome/autophagosome ratio, and decreases p62	[117]
Apoptosis	HDAC2	Promotes apoptosis via downregulating BMP-7 epigenetically	[30]
	HDAC5	Reduces cisplatin-mediated activation of some caspases, thereby inhibiting apoptosis	[32]
	HDAC6	Inhibits the expression of E-cadherin and improves ERS	[37, 38]
	SIRT1	Suppresses the acetylation of p53, and thus inhibits the apoptotic pathway	[52]
	SIRT2	Decreases the acetylation of MKP-1, thereby increasing the phosphorylation of p38 and c-Jun	[56]
	SIRT5	Upregulates Nrf2, HO-1, and Bcl-2 expression, reduces cytochrome c release, thereby inhibiting apoptosis progress	[76]
	SIRT6	Inhibits ERK1/2 through histone H3K9 deacetylation	[81]
Inflammatory response	HDAC6	Increases abnormal phosphorylation/activation of NF-κB	[37]
	SIRT1	Reduces macrophage infiltration; increases acetylation of p65 and subsequent NF-κB activation, thereby increasing pro-inflammatory cytokines	[49, 53]
	SIRT3	Suppresses the nuclear translocation of NF-κB and inflammatory cell infiltration	[66, 67]
	SIRT4	Inhibits NF-κB signaling and the NLRP3 inflammasome	[73]
	SIRT7	Limits the nuclear translocation of phospho-p65 and reduces the secretion of inflammatory cytokines	[86, 87]

HDAC: histone deacetylases; AKI: acute kidney injury; LKB1: liver kinase B1; AMPK: AMP-activated protein kinase; DRP1: dynamin-related protein 1; MFF: mitochondrial fission factor; SOD: superoxide dismutase; ROS: reactive oxygen species; Nrf2: nuclear factor red lineage 2-related factor 2; Atg7: autophagy related protein 7; BMP-7: morphogenetic protein 7; ERS: endoplasmic reticulum stress; MKP-1: mitogen-activated protein kinase phosphatase-1; HO-1: heme oxygenase-1; Bcl-2, B-cell lymphoma 2; ERK1/2: extracellular signal-regulated kinases; NF-κB: factor-kappaB.

## Clinical translation and therapeutic implications of HDAC inhibitors and SIRT activators

The aforementioned findings have provided compelling evidence for the potential efficacy of HDACs against cisplatin-induced nephrotoxicity. Therefore, the utilization of HDAC inhibitors or inducers might further augment their renal protection during cisplatin chemotherapy. Numerous natural substances and synthetic agents have been validated to regulate the expression or activity of HDACs to treat cisplatin-induced nephrotoxicity while optimizing the therapeutic efficacy of cisplatin [89–100] (Table 2).

**Table 2.** The renoprotective function of HDAC specific modulators in cisplatin-induced nephrotoxicity.

Target	Compound	Effects on cisplatin-induced AKI	Effect of HDACs modulators on cisplatin’s chemosensitivity	Refs.
HDAC2 inhibitor	18βGA	Inhibits renal tubular epithelial cells apoptosis via enhancing BMP-7 epigenetically	Combining glycyrrhizin and cisplatin reverses the cisplatin resistance in hepatocellular carcinoma cells through the inhibition of multidrug resistance-associated proteins	[90, 107]
HDAC6 inhibitor	TA	Suppresses tubular injury, inhibits oxidative stress, and reduces the inflammatory response	Combining TA and cisplatin overcomes cisplatin chemoresistance by targeting cancer stem cells in oral squamous cell carcinoma	[37, 98]
	23BB	Regulates tubular epithelial cell apoptosis via the inactivation of ERS	Combining 23BB and cisplatin produces synergistic anticancer effects in colorectal HCT116, acute myelocytic leukemia MV4-11, and B cell lymphoma Romas xenografts	[38, 93]
Class I inhibitor	VPA	Promotes expression of BMP-7 and inhibits apoptosis	Enhances cisplatin sensitivity of non-small cell lung cancer cells	[30, 94]
	MS-275	Reduces inflammation, apoptosis, and cell proliferation	MS-275 combined with cisplatin exerts synergistic antitumour effects in human esophageal squamous cell carcinoma cells	[96]
Class I/II inhibitor	βOHB	Ameliorates renal tubular cell death in cisplatin treatment	βOHB enhances cisplatin-induced apoptosis in hepatocellular carcinoma cells and exerts a synergistic effect for cisplatin chemotherapy.	[32, 97]
	TSA	Activates autophagy and inhibits apoptosis and inflammatory response	Combining histone deacetylase inhibitors and cisplatin produce synergistic anticancer effects in breast cancer and cholangiocarcinoma	[89, 92, 104, 105]
	SAHA	Activates autophagy	Combining histone deacetylase inhibitors and cisplatin produce synergistic anticancer effects in breast cancer and cholangiocarcinoma	[89, 92, 105]
SIRT1 activator	SRT1720	Suppresses apoptosis, oxidative stress, and inflammation	Augmentes the antitumour effects of cisplatin in a panel of NSCLC	[49, 91]
	Resveratrol	Reduces p53 acetylation and inhibits the apoptotic pathway	Co-administration of Res and cisplatin can prevent chemoresistance and potentiate the induction of apoptosis and cell cycle arrest in cancer cells	[52, 99]
SIRT3 activator	Curcumin	Recovers SIRT3 level to protect mitochondria damage	Co-treatment with curcumin and cisplatin enhances the synergistic effect and thus inhibits cancers such as prostate, hepatocellular, gastric, Hodgkin lymphoma, bladder, and colorectal.	[62]
	Silybin	Promotes cell regeneration and improves mitochondrial function	Silybin combined with cisplatin exerts an antiproliferative effect on gynecological malignancies	[63]
	Matrine	Activates the SIRT3/OPA1 axis and improves mitochondrial function	The combination of matrine and cisplatin may promote tumour cell death in liver and bladder cancer	[67]
	Honokiol	Inhibits mitochondrial fission	Combining Honokiol and cisplatin plays a synergistic antitumour role in lung cancer models	[68, 100]
SIRT6 activator	Isoorientin	Decreases oxidative stress	Reverses lung cancer drug resistance by promoting ferroptosis via the SIRT6/Nrf2/GPX4 signaling pathway	[84]
	Polydatin	Activates autophagy and protects against AKI	Polydatin and Cisplatin in combination synergistically reduce tumour size and inhibit lymph node metastasis in vivo model of oral cancer	[13]

HDAC: histone deacetylases; AKI: acute kidney injury; GA: glycyrrhizic acid; BMP-7: morphogenetic protein 7; TA: Tubastatin A; ERS: endoplasmic reticulum stress; VPA: valproic acid; βOHB: β-hydroxybutyrate; TSA: trichostatin A; SAHA: suberoylanilide hydroxamic acid; NSCLC: non-small cell lung carcinoma; Bcl-2, B-cell lymphoma 2; Bax: Bcl-2-associated X protein; OPA1: optic atrophy 1; FAO: fatty acid oxidation; n/a: not assessed.

## HDACi as nephroprotective drugs in cisplatin nephrotoxicity

HDAC inhibitors (HDACi) have been primarily studied and used in the treatment of various types of cancer [101]. Nevertheless, recent research has shown that inhibitors of the Zn2+-dependent HDACs, such as hydroxamic acids, short-chain fatty acids (SCFAs), benzamides, electrophilic ketones, and cyclic peptides possess protective properties in cisplatin-induced AKI as well [102]. Trichostatin A (TSA) and suberoylanilide hydroxamic acid (SAHA), classified as hydroxamic acids, were initially discovered for their capacity to elicit cell cycle arrest and differentiation in cancer cells [103]. Later on, their broad-spectrum inhibitory effects on class I and II HDACs were uncovered. Previous studies found that treatment with TSA could activate microglia/macrophage WAP domain protein (AMWAP) and increase its expression in RTECs, exerting an anti-inflammatory and anti-apoptotic function [104]. Furthermore, SAHA and TSA can enhance the autophagy activity of proximal RTECs through the AMPK/mTOR signaling pathway, whereas knockout of autophagy-related genes could weaken their renal protective effect, suggesting that HDACi play a renal protective role by enhancing autophagy [105]. While valproic acid (VPA), an inhibitor of the SCFAs, exhibits moderate potency and a certain degree of selectivity towards class I HDACs. It is targeted at HDAC2/BMP-7 axis to inhibit apoptosis of RTECs [30]. Consistently, tubastatin A (TA), a benzamide compound that exhibits remarkable selectivity towards HDAC6, safeguards against kidney injury by various HDAC6-mediated mechanisms, including the inhibition of apoptosis and inflammation, and the induction of autophagy [37]. Compound 23BB is another highly selective inhibitor of HDAC6, mechanistically, weakening the HDAC6 activity via enhancing histone H3 acetylation. Inhibition of HDAC6 by 23BB modulates cisplatin-induced apoptosis of RTECs through suppressing ERS [38].

Besides, several natural products also inhibit HDAC expression or activity and relieve kidney damage. Significantly, these natural products exhibit a multitude of advantages in comparison to conventional chemical compound-based medications, encompassing fewer adverse effects and variable bioavailability. Glycyrrhizic acid (GA), the principal bioactive constituent found in Licorice, exhibits a broad spectrum of pharmacological effects, including anti-tumour and anti-inflammatory properties [106]. A particular study revealed that the renoprotective effects of 18βGA are attributed to its ability to inhibit apoptosis in RTECs, which is achieved by restoring BMP-7 signaling through epigenetic regulation, specifically via a mechanism dependent on HDAC2 [107]. β-hydroxybutyrate (βOHB), a ketone body produced during the oxidation of fatty acids, has been identified as a class I and II HDAC inhibitor under normal conditions [108]. Nevertheless, in cisplatin-induced AKI models, it has been found that βOHB remarkably enhances the expression of HDAC5 and keeps it within the nucleus, reducing the destructive effects of cisplatin on DNA and apoptosis, and ameliorating kidney damage [32]. Hence, it could be speculated that the protective efficacy of βOHB, whether as an inhibitor or activator of HDACs, may rely on experimental circumstances and the specific HDAC subtype targeted by βOHB.

## Sirtuins-specific modulators as nephroprotective drugs in cisplatin nephrotoxicity

The investigation of sirtuins has unveiled numerous protein targets implicated in the development of cisplatin nephrotoxicity, and specific sirtuins have demonstrated robust renoprotective effects. Therefore, modulators that specifically target sirtuins offer a clinical approach to treating kidney disease. Extensive research has substantiated the therapeutic effectiveness of small molecules in modulating the activity of sirtuins for cisplatin-mediated nephrotoxicity.

SRT1720 is a synthetic polyphenolic compound renowned for its potent and selective targeting of SIRT1 [109]. It can activate SIRT1 to inhibit oxidative stress, inflammation, and apoptosis, which attenuates cisplatin-induced nephrotoxicity [49]. Resveratrol, a polyphenol that is abundant in grape skin and seeds, has been shown to alleviate acetylation of p53 induced by cisplatin, and apoptosis, as well as mitigate apoptosis and cytotoxicity in RTECs. According to research findings, the activation of SIRT1 by resveratrol has exhibited a reduction in cisplatin-induced apoptosis, coupled with an enhancement in the glomerular filtration rate [52]. These findings further support the notion that pharmacologically activating SIRT1 could serve as a promising therapeutic approach in combating cisplatin-induced kidney damage.

Curcumin and Silybin are both well-known natural agonists of SIRT3, which can prevent the mitochondrial ultrastructure instability caused by cisplatin-induced nephrotoxicity and disturb mitochondrial dynamics respectively, again verifying the conclusion that SIRT3 has a protective effect on cisplatin-induced mitochondrial damage [62,63]. Matrine (MAT), a tetracyclic quinolizine alkaloid derived from the Sophora genus, exhibits a multitude of pharmacological effects, encompassing its remarkable capacity to counteract oxidative stress, inflammation, and apoptosis [110]. Studies have convincingly shown that the renal protective effects of MAT are closely linked to the enhancement of mitochondrial function through activation of the SIRT3/OPA1 pathway [67]. Honokiol, a major bioactive compound extracted from Magnolia, has been reported to exhibit notable effects in cisplatin-induced AKI models. Specifically, it can activate SIRT3, consequently preventing mitochondrial fragmentation and subsequent cell injury and death [60]. Undoubtedly, SIRT3, serving as a pivotal modulator of mitochondrial dynamics, effectively shielded against tubular injury in animal models of cisplatin nephrotoxicity and emerged as a prospective target for enhancing the prognosis of cisplatin-induced kidney injury.

Isoorientin, a flavonoid-like compound, decreases oxidative stress in cisplatin-induced AKI by activating the SIRT1/SIRT6-Nrf2 pathway [84]. Polydatin (PD), a glucoside of resveratrol that is naturally extracted from Polygonum, is widely used in both medication and food and has a multitarget protective effect in AKI [111,112]. It has been disclosed that enhances autophagy flux by upregulating SIRT6, which alleviates cisplatin-induced nephrotoxicity [113].

Consequently, sirtuins-specific activators may assume an active therapeutic role in the prevention and treatment of nephrotoxicity. Diverging from the remaining sirtuins, deficiencies in SIRT2 and SIRT7 exhibit a nephroprotective effect. Bombesin receptor-activated protein acts as a ligand for an orphan G-protein-coupled receptor that attenuates proteasomal degradation of SIRT2, this event subsequently triggers the activation of the NF-κB subunit p65 and enhances RTECs apoptosis in cisplatin-induced nephrotoxicity [114].

These pre-clinical findings reveal crucial clinical therapeutic implications for HDAC inhibitors and SIRT activators in cisplatin-induced AKI. However, as some compounds have multiple targets, they manifest relatively low selectivity, causing untoward effects like fatigue, nausea, and some cardiotoxicity. The clinical translation of nephroprotective approaches for cisplatin-induced AKI remains challenging. Thus, the renal-specific delivery of these inhibitors or activators is essential to acquire their renoprotective effects. Pre-clinical studies have shown that nanoparticle-based drug delivery platforms can be used to target therapeutic agents to diseased kidney tissue [115]. Moreover, advancements in molecular biology techniques have led to performing research on targeted therapy using natural products or derivatives that are highly selective for the kidney as carriers, chemically coupling these factors into biological therapy [116]. All of these open up new ideas for protective measures against cisplatin-induced nephrotoxicity.

## HDACs and nephrotoxicity due to other chemotherapy agents

In addition to playing a role in cisplatin-induced nephrotoxicity, HDACs have also been found to alleviate nephrotoxicity caused by other types of chemotherapeutic agents. L-carnitine diminished oxidative stress, renal inflammation, and apoptosis by controlling the expression of renal SIRT1/PGC-1/Nrf2/HO-1 axis, protecting against methotrexate (MTX)-induced nephrotoxicity [117]. Curcumin has been found to attenuate MTX-induced nephrotoxicity through its antioxidant and anti-inflammatory properties by preserving GSH and SOD activities and inhibiting TNF-α and cyclooxygenase-2 (COX-2) production [118]. Phenethyl isothiocyanate prevented cyclophosphamide-induced nephrotoxicity by decreasing oxidative damage through Nrf2 and SIRT1 activation and NF-κB inhibition [119]. Acylated ghrelin significantly increased the levels and activity of SIRT1, thereby reducing the deacetylation of p53 and NF-κB, which prevented doxorubicin-induced nephropathy [120]. Compared with cisplatin, HDACs have been less studied to protect against kidney damage in other types of chemotherapy drugs. However, these studies not only illustrate the extensive and comprehensive role of HDACs in nephrotoxicity caused by anti-chemotherapy drugs but also reflect the feasibility and safety of combining HDAC modifiers with cisplatin and/or other types of chemotherapeutic drugs.

## Conclusion and future perspectives

Renal function impairment and nephrotoxicity are one of the major limitations of cancer treatment. Within the scope of onconephrology, heightened focus on the bidirectional relation between chemotherapeutic and renal damage is imperative. The underlying mechanisms of cisplatin-induced nephrotoxicity encompass mitochondrial injury, oxidative stress, autophagy, apoptosis, and inflammation. The participation of HDACs in the pathogenesis of cisplatin-induced nephrotoxicity underscores their potential as therapeutic targets for ameliorating renal damage. However, whether HDAC modulators could both augment the therapeutic effects against cancer cells and safeguard normal cells in the kidneys of tumour-bearing animals, is yet to be determined. Clearly, adequately powered clinical trials and retrospective or prospective analysis of hard endpoints are urgently needed in this regard. Alternatively, cisplatin has multiple targets in cells, so abrogating singular injury processes may confer only a partial nephroprotective effect. Meanwhile, the specificity of HDAC modulation is wanting, making it difficult to effectively monitor regulated genes. Novel HDAC modulators, therefore, need to be developed in two directions, one is a multi-target-coupled HDAC regulator, and the other is a strongly selective HDAC regulator. Overall, targeting HDACs may offer new avenues for preventing or treating cisplatin nephrotoxicity and improving patient outcomes.

## Data Availability

All figures and tables are original and are not taken from other publications. Data sharing is not applicable to this article, as no new data were created or analysed in this study.

## References

[1] Romani AMP. Cisplatin in cancer treatment. Biochem Pharmacol. 2022;206:115323. doi: 10.1016/j.bcp.2022.115323. 36368406

[2] de Brito RV, Mancini MW, Palumbo MDN, et al. The rationale for "laser-induced thermal therapy (LITT) and intratumoral cisplatin" approach for cancer treatment. Int J Mol Sci. 2022;23(11):5934. doi: 10.3390/ijms23115934. PMC9180481 35682611

[3] Pabla N, Dong Z. Cisplatin nephrotoxicity: mechanisms and renoprotective strategies. Kidney Int. 2008;73(9):994–1007. doi: 10.1038/sj.ki.5002786. 18272962

[4] Tang C, Livingston MJ, Safirstein R, et al. Cisplatin nephrotoxicity: new insights and therapeutic implications. Nat Rev Nephrol. 2023;19(1):53–72. doi: 10.1038/s41581-022-00631-7. 36229672

[5] Crona DJ, Faso A, Nishijima TF, et al. A systematic ­review of strategies to prevent cisplatin-induced nephrotoxicity. Oncologist. 2017;22(5):609–619. doi: 10.1634/theoncologist.2016-0319. 28438887 PMC5423518

[6] Volarevic V, Djokovic B, Jankovic MG, et al. Molecular mechanisms of cisplatin-induced nephrotoxicity: a balance on the knife edge between renoprotection and tumor toxicity. J Biomed Sci. 2019;26(1):25. doi: 10.1186/s12929-019-0518-9. 30866950 PMC6417243

[7] Choudhary C, Kumar C, Gnad F, et al. Lysine acetylation targets protein complexes and co-regulates major cellular functions. Science. 2009;325(5942):834–840. doi: 10.1126/science.1175371. 19608861

[8] Moreno-Yruela C, Bæk M, Monda F, et al. Chiral posttranslational modification to lysine ε-amino groups. Acc Chem Res. 2022;55(10):1456–1466. doi: 10.1021/acs.accounts.2c00115. 35500056

[9] Dang F, Wei W. Targeting the acetylation signaling pathway in cancer therapy. Semin Cancer Biol. 2022;85:209–218. doi: 10.1016/j.semcancer.2021.03.001. 33705871 PMC8423867

[10] Fan X, Wei W, Huang J, et al. Daphnetin attenuated cisplatin-induced acute nephrotoxicity with enhancing antitumor activity of cisplatin by upregulating SIRT1/SIRT6-Nrf2 pathway. Front Pharmacol. 2020;11:579178. doi: 10.3389/fphar.2020.579178. 33363464 PMC7753212

[11] Wei H, Gou W, Gao J, et al. Novel PHD2/HDACs hybrid inhibitors protect against cisplatin-induced acute kidney injury. Eur J Med Chem. 2022;230:114115. doi: 10.1016/j.ejmech.2022.114115. 35033824

[12] Moran B, Davern M, Reynolds JV, et al. The impact of histone deacetylase inhibitors on immune cells and implications for cancer therapy. Cancer Lett. 2023;559:216121. doi: 10.1016/j.canlet.2023.216121. 36893893

[13] Ruzic D, Djokovic N, Srdic-Rajic T, et al. Targeting histone deacetylases: opportunities for cancer treatment and chemoprevention. Pharmaceutics. 2022;14(1):209. doi: 10.3390/pharmaceutics14010209. PMC8778744 35057104

[14] Fujishiro H, Taguchi H, Hamao S, et al. Comparisons of segment-specific toxicity of platinum-based agents and cadmium using S1, S2, and S3 cells derived from mouse kidney proximal tubules. Toxicol In Vitro. 2021;75:105179. doi: 10.1016/j.tiv.2021.105179. 33905841

[15] Pabla N, Murphy RF, Liu K, et al. The copper transporter Ctr1 contributes to cisplatin uptake by renal tubular cells during cisplatin nephrotoxicity. Am J Physiol Renal Physiol. 2009;296(3):F505–11. doi: 10.1152/ajprenal.90545.2008. 19144690 PMC2660190

[16] Eltayeb SA, Ciarimboli G, Beul K, et al. Role of organic cation transporter 2 in autophagy induced by platinum derivatives. Int J Mol Sci. 2022;23(3):1090. doi: 10.3390/ijms23031090. PMC8834759 35163014

[17] Tang C, Cai J, Yin XM, et al. Mitochondrial quality control in kidney injury and repair. Nat Rev Nephrol. 2021;17(5):299–318. doi: 10.1038/s41581-020-00369-0. 33235391 PMC8958893

[18] Wang S, Chen Y, Wu H, et al. Role of Transcription Factor EB in Mitochondrial Dysfunction of Cisplatin-Induced Acute Kidney Injury. Int J Mol Sci. 2023;24(3):3028. doi: 10.3390/ijms24033028. PMC9917568 36769347

[19] Yu X, Meng X, Xu M, et al. Celastrol ameliorates cisplatin nephrotoxicity by inhibiting NF-κB and improving mitochondrial function. EBioMedicine. 2018;36:266–280. doi: 10.1016/j.ebiom.2018.09.031. 30268831 PMC6197337

[20] Chirino YI, Pedraza-Chaverri J. Role of oxidative and nitrosative stress in cisplatin-induced nephrotoxicity. Exp Toxicol Pathol. 2009;61(3):223–242. doi: 10.1016/j.etp.2008.09.003. 18986801

[21] Ni J, Hou X, Wang X, et al. 3-Deazaneplanocin A protects against cisplatin-induced renal tubular cell apoptosis and acute kidney injury by restoration of E-cadherin expression. Cell Death Dis. 2019;10(5):355. doi: 10.1038/s41419-019-1589-y. 31043583 PMC6494881

[22] Liu H, Baliga R. Endoplasmic reticulum stress-associated caspase 12 mediates cisplatin-induced LLC-PK1 cell apoptosis. J Am Soc Nephrol. 2005;16(7):1985–1992. doi: 10.1681/ASN.2004090768. 15901768

[23] Tsuruya K, Ninomiya T, Tokumoto M, et al. Direct involvement of the receptor-mediated apoptotic pathways in cisplatin-induced renal tubular cell death. Kidney Int. 2003;63(1):72–82. doi: 10.1046/j.1523-1755.2003.00709.x. 12472770

[24] Benedetti G, Fredriksson L, Herpers B, et al. TNF-alpha-mediated NF-kappaB survival signaling impairment by cisplatin enhances JNK activation allowing synergistic apoptosis of renal proximal tubular cells. Biochem Pharmacol. 2013;85(2):274–286. doi: 10.1016/j.bcp.2012.10.012. 23103562

[25] Holbrook J, Lara-Reyna S, Jarosz-Griffiths H, et al. Tumour necrosis factor signalling in health and disease. F1000Res. 2019;8:111. doi: 10.12688/f1000research.17023.1. PMC6352924 30755793

[26] Gong L, Pan Q, Yang N. Autophagy and inflammation regulation in acute kidney injury. Front Physiol. 2020;11:576463. doi: 10.3389/fphys.2020.576463. 33101057 PMC7546328

[27] Inoue K, Kuwana H, Shimamura Y, et al. Cisplatin-induced macroautophagy occurs prior to apoptosis in proximal tubules *in vivo*. Clin Exp Nephrol. 2010;14(2):112–122. doi: 10.1007/s10157-009-0254-7. 20013139

[28] Hagelkruys A, Mattes K, Moos V, et al. Essential nonredundant function of the catalytic activity of histone deacetylase 2 in mouse development. Mol Cell Biol. 2016;36(3):462–474. doi: 10.1128/MCB.00639-15. 26598605 PMC4719423

[29] Hyndman KA, Crossman DK. Kidney cell type-specific changes in the chromatin and transcriptome landscapes following epithelial Hdac1 and Hdac2 knockdown. Physiol Genomics. 2022;54(2):45–57. doi: 10.1152/physiolgenomics.00102.2021. 34890513 PMC8791845

[30] Ma T, Huang C, Xu Q, et al. Suppression of BMP-7 by histone deacetylase 2 promoted apoptosis of renal tubular epithelial cells in acute kidney injury. Cell Death Dis. 2017;8(10):e3139–e3139. doi: 10.1038/cddis.2017.552. 29072686 PMC5680919

[31] Xu Z, Jia K, Wang H, et al. METTL14-regulated PI3K/Akt signaling pathway via PTEN affects HDAC5-mediated epithelial-mesenchymal transition of renal tubular cells in diabetic kidney disease. Cell Death Dis. 2021;12(1):32. doi: 10.1038/s41419-020-03312-0. 33414476 PMC7791055

[32] Mikami D, Kobayashi M, Uwada J, et al. beta-Hydroxybutyrate, a ketone body, reduces the cytotoxic effect of cisplatin via activation of HDAC5 in human renal cortical epithelial cells. Life Sci. 2019;222:125–132. doi: 10.1016/j.lfs.2019.03.008. 30851335

[33] Hubbert C, Guardiola A, Shao R, et al. HDAC6 is a microtubule-associated deacetylase. Nature. 2002;417(6887):455–458. doi: 10.1038/417455a. 12024216

[34] Li Y, Shin D, Kwon SH. Histone deacetylase 6 plays a role as a distinct regulator of diverse cellular processes. FEBS J. 2013;280(3):775–793. doi: 10.1111/febs.12079. 23181831

[35] Shi L, Song Z, Li C, et al. HDAC6 inhibition alleviates ischemia- and cisplatin-induced acute kidney injury by promoting autophagy. Cells. 2022;11(24):3951. doi: 10.3390/cells11243951. PMC9776591 36552715

[36] Kumar S, Park SH, Cieply B, et al. A pathway for the control of anoikis sensitivity by E-cadherin and epithelial-to-mesenchymal transition. Mol Cell Biol. 2011;31(19):4036–4051. doi: 10.1128/MCB.01342-10. 21746881 PMC3187352

[37] Tang J, Shi Y, Liu N, et al. Blockade of histone deacetylase 6 protects against cisplatin-induced acute kidney injury. Clin Sci. 2018;132(3):339–359. doi: 10.1042/CS20171417. 29358506

[38] Hao Y, Guo F, Huang Z, et al. 2-Methylquinazoline derivative 23BB as a highly selective histone deacetylase 6 inhibitor alleviated cisplatin-induced acute kidney injury. Biosci Rep. 2020;40(1):BSR20191538. doi: 10.1042/BSR20191538. PMC6970081 31894849

[39] Boyce M, Bryant KF, Jousse C, et al. A selective inhibitor of eIF2alpha dephosphorylation protects cells from ER stress. Science. 2005;307(5711):935–939. doi: 10.1126/science.1101902. 15705855

[40] Li HF, Cheng CF, Liao WJ, et al. ATF3-mediated epigenetic regulation protects against acute kidney injury. J Am Soc Nephrol. 2010;21(6):1003–1013. doi: 10.1681/ASN.2009070690. 20360311 PMC2900964

[41] Hyndman KA, Kasztan M, Mendoza LD, et al. Dynamic changes in histone deacetylases following kidney ischemia-reperfusion injury are critical for promoting proximal tubule proliferation. Am J Physiol Renal Physiol. 2019;316(5):F875–F888. doi: 10.1152/ajprenal.00499.2018. 30810062 PMC6580243

[42] Machiela E, Liontis T, Dues DJ, et al. Disruption of mitochondrial dynamics increases stress resistance through activation of multiple stress response pathways. Faseb J. 2020;34(6):8475–8492. doi: 10.1096/fj.201903235R. 32385951 PMC7313680

[43] Ha SD, Solomon O, Akbari M, et al. Histone deacetylase 8 protects human proximal tubular epithelial cells from hypoxia-mimetic cobalt- and hypoxia/reoxygenation-induced mitochondrial fission and cytotoxicity. Sci Rep. 2018;8(1):11332. doi: 10.1038/s41598-018-29463-x. 30054507 PMC6063935

[44] Kim JI, Jung KJ, Jang HS, et al. Gender-specific role of HDAC11 in kidney ischemia- and reperfusion-induced PAI-1 expression and injury. Am J Physiol Renal Physiol. 2013;305(1):F61–70. doi: 10.1152/ajprenal.00015.2013. 23657855

[45] Chen L, Wang Z, Xu Q, et al. The failure of DAC to induce OCT2 expression and its remission by hemoglobin-based nanocarriers under hypoxia in renal cell carcinoma. Theranostics. 2020;10(8):3562–3578. doi: 10.7150/thno.39944. 32206108 PMC7069078

[46] Zhu Q, Yu L, Qin Z, et al. Regulation of OCT2 transcriptional repression by histone acetylation in renal cell carcinoma. Epigenetics. 2019;14(8):791–803. doi: 10.1080/15592294.2019.1615354. 31088315 PMC6615535

[47] Tovar-Palacio C, Noriega LG, Mercado A. Potential of polyphenols to restore SIRT1 and NAD+ metabolism in renal disease. Nutrients. 2022;14(3):653. doi: 10.3390/nu14030653. PMC8837945 35277012

[48] Hasegawa K, Wakino S, Yoshioka K, et al. Kidney-specific overexpression of Sirt1 protects against acute kidney injury by retaining peroxisome function. J Biol Chem. 2010;285(17):13045–13056. doi: 10.1074/jbc.M109.067728. 20139070 PMC2857112

[49] Kim JY, Jo J, Kim K, et al. Pharmacological activation of Sirt1 ameliorates cisplatin-induced acute kidney injury by suppressing apoptosis, oxidative stress, and inflammation in mice. Antioxidants. 2019;8(8):322. doi: 10.3390/antiox8080322. PMC6720310 31431003

[50] Lee CG, Kim JG, Kim HJ, et al. Discovery of an integrative network of microRNAs and transcriptomics changes for acute kidney injury. Kidney Int. 2014;86(5):943–953. doi: 10.1038/ki.2014.117. 24759152

[51] Vaziri H, Dessain SK, Ng Eaton E, et al. hSIR2(SIRT1) functions as an NAD-dependent p53 deacetylase. Cell. 2001;107(2):149–159. doi: 10.1016/s0092-8674(01)00527-x. 11672523

[52] Kim DH, Jung YJ, Lee JE, et al. SIRT1 activation by resveratrol ameliorates cisplatin-induced renal injury through deacetylation of p53. Am J Physiol Renal Physiol. 2011;301(2):F427–35. doi: 10.1152/ajprenal.00258.2010. 21593185

[53] Han S, Lin F, Ruan Y, et al. miR-132-3p promotes the cisplatin-induced apoptosis and inflammatory response of renal tubular epithelial cells by targeting SIRT1 via the NF-kappaB pathway. Int Immunopharmacol. 2021;99:108022. doi: 10.1016/j.intimp.2021.108022. 34339961

[54] Fu Y, Wang Y, Liu Y, et al. p53/Sirtuin 1/NF-kappaB signaling axis in chronic inflammation and maladaptive kidney repair after cisplatin nephrotoxicity. Front Immunol. 2022;13:925738. doi: 10.3389/fimmu.2022.925738. 35874713 PMC9301469

[55] Jung YJ, Lee AS, Nguyen-Thanh T, et al. SIRT2 regulates LPS-induced renal tubular CXCL2 and CCL2 expression. J Am Soc Nephrol. 2015;26(7):1549–1560. doi: 10.1681/ASN.2014030226. 25349202 PMC4483578

[56] Jung YJ, Park W, Kang KP, et al. SIRT2 is involved in cisplatin-induced acute kidney injury through regulation of mitogen-activated protein kinase phosphatase-1. Nephrol Dial Transplant. 2020;35(7):1145–1156. doi: 10.1093/ndt/gfaa042. 32240312

[57] Zhang J, Xiang H, Liu J, et al. Mitochondrial Sirtuin 3: new emerging biological function and therapeutic target. Theranostics. 2020;10(18):8315–8342. doi: 10.7150/thno.45922. 32724473 PMC7381741

[58] Yuan J, Zhao J, Qin Y, et al. The protective mechanism of SIRT3 and potential therapy in acute kidney injury. QJM Month J Assoc Phys. 2024;117(4):247–255. doi:10.1093/qjmed/hcad152. 37354530

[59] Wang X, Zhu H, Hu J, et al. Magnesium isoglycyrrhizinate reduces the target-binding amount of cisplatin to mitochondrial DNA and renal injury through SIRT3. Int J Mol Sci. 2022;23(21):13093. doi: 10.3390/ijms232113093. PMC9654254 36361883

[60] Mao RW, He SP, Lan JG, et al. Honokiol ameliorates cisplatin-induced acute kidney injury via inhibition of mitochondrial fission. Br J Pharmacol. 2022;179(14):3886–3904. doi: 10.1111/bph.15837. 35297042

[61] Morigi M, Perico L, Rota C, et al. Sirtuin 3-dependent mitochondrial dynamic improvements protect against acute kidney injury. J Clin Invest. 2015;125(2):715–726. doi: 10.1172/JCI77632. 25607838 PMC4319434

[62] Li Y, Ye Z, Lai W, et al. Activation of sirtuin 3 by silybin attenuates mitochondrial dysfunction in cisplatin-induced acute kidney injury. Front Pharmacol. 2017;8:178. doi: 10.3389/fphar.2017.00178. 28424621 PMC5380914

[63] Ortega-Domínguez B, Aparicio-Trejo OE, García-Arroyo FE, et al. Curcumin prevents cisplatin-induced renal alterations in mitochondrial bioenergetics and dynamic. Food Chem Toxicol. 2017;107(Pt A):373–385. doi: 10.1016/j.fct.2017.07.018. 28698153

[64] Perico L, Morigi M, Benigni A. Mitochondrial sirtuin 3 and renal diseases. Nephron. 2016;134(1):14–19. doi: 10.1159/000444370. 27362524

[65] Yoon SP, Kim J. Poly(ADP-ribose) polymerase 1 contributes to oxidative stress through downregulation of sirtuin 3 during cisplatin nephrotoxicity. Anat Cell Biol. 2016;49(3):165–176. doi: 10.5115/acb.2016.49.3.165. 27722009 PMC5052225

[66] Kim D, Park W, Lee S, et al. Absence of Sirt3 aggravates cisplatin nephrotoxicity via enhanced renal tubular apoptosis and inflammation. Mol Med Rep. 2018;18(4):3665–3672. doi: 10.3892/mmr.2018.9350. 30106119 PMC6131565

[67] Yuan L, Yang J, Li Y, et al. Matrine alleviates cisplatin-induced acute kidney injury by inhibiting mitochondrial dysfunction and inflammation via SIRT3/OPA1 pathway. J Cell Mol Med. 2022;26(13):3702–3715. doi: 10.1111/jcmm.17398. 35650472 PMC9258713

[68] Li M, Li C-M, Ye Z-C, et al. Sirt3 modulates fatty acid oxidation and attenuates cisplatin-induced AKI in mice. J Cell Mol Med. 2020;24(9):5109–5121. doi: 10.1111/jcmm.15148. 32281286 PMC7205836

[69] Du P, Liu T, Luo P, et al. SIRT3/GLUT4 signaling activation by metformin protect against cisplatin-induced ototoxicity *in vitro*. Arch Toxicol. 2023;97(4):1147–1162. doi: 10.1007/s00204-023-03457-9. 36800006

[70] Han Y, Zhou S, Coetzee S, et al. SIRT4 and its roles in energy and redox metabolism in health, disease and during exercise. Front Physiol. 2019;10:1006. doi: 10.3389/fphys.2019.01006. 31447696 PMC6695564

[71] Haigis MC, Mostoslavsky R, Haigis KM, et al. SIRT4 inhibits glutamate dehydrogenase and opposes the effects of calorie restriction in pancreatic beta cells. Cell. 2006;126(5):941–954. doi: 10.1016/j.cell.2006.06.057. 16959573

[72] Ugur S, Ulu R, Dogukan A, et al. The renoprotective effect of curcumin in cisplatin-induced nephrotoxicity. Ren Fail. 2015;37(2):332–336. doi: 10.3109/0886022X.2014.986005. 25594614

[73] Xu X, Zhang L, Hua F, et al. FOXM1-activated SIRT4 inhibits NF-kappaB signaling and NLRP3 inflammasome to alleviate kidney injury and podocyte pyroptosis in diabetic nephropathy. Exp Cell Res. 2021;408(2):112863. doi: 10.1016/j.yexcr.2021.112863. 34626587

[74] Tan M, Peng C, Anderson KA, et al. Lysine glutarylation is a protein posttranslational modification regulated by SIRT5. Cell Metab. 2014;19(4):605–617. doi: 10.1016/j.cmet.2014.03.014. 24703693 PMC4108075

[75] Du J, Zhou Y, Su X, et al. Sirt5 is a NAD-dependent protein lysine demalonylase and desuccinylase. Science. 2011;334(6057):806–809. doi: 10.1126/science.1207861. 22076378 PMC3217313

[76] Li W, Yang Y, Li Y, et al. Sirt5 attenuates cisplatin-induced acute kidney injury through regulation of Nrf2/HO-1 and Bcl-2. Biomed Res Int. 2019;2019:4745132–4745111. doi: 10.1155/2019/4745132. 31815138 PMC6878818

[77] Chiba T, Peasley KD, Cargill KR, et al. Sirtuin 5 regulates proximal tubule fatty acid oxidation to protect against AKI. J Am Soc Nephrol. 2019;30(12):2384–2398. doi: 10.1681/ASN.2019020163. 31575700 PMC6900790

[78] Gewin LS. Sirtuin 6 and renal injury: another link in the beta-catenin chain? Kidney Int. 2020;97(1):24–27. doi: 10.1016/j.kint.2019.09.022. 31901350

[79] Lu Z, Xu S. ERK1/2 MAP kinases in cell survival and apoptosis. IUBMB Life. 2006;58(11):621–631. doi: 10.1080/15216540600957438. 17085381

[80] Kwon H-J, Choi G-E, Ryu S, et al. Stepwise phosphorylation of p65 promotes NF-κB activation and NK cell responses during target cell recognition. Nat Commun. 2016;7(1):11686. doi: 10.1038/ncomms11686. 27221592 PMC4894962

[81] Li Z, Xu K, Zhang N, et al. Overexpressed SIRT6 attenuates cisplatin-induced acute kidney injury by inhibiting ERK1/2 signaling. Kidney Int. 2018;93(4):881–892. doi: 10.1016/j.kint.2017.10.021. 29373150

[82] Huang Y-C, Tsai M-S, Hsieh P-C, et al. Galangin ameliorates cisplatin-induced nephrotoxicity by attenuating oxidative stress, inflammation and cell death in mice through inhibition of ERK and NF-kappaB signaling. Toxicol Appl Pharmacol. 2017;329:128–139. doi: 10.1016/j.taap.2017.05.034. 28558962

[83] Zhang W, Wei R, Zhang L, et al. Sirtuin 6 protects the brain from cerebral ischemia/reperfusion injury through NRF2 activation. Neuroscience. 2017;366:95–104. doi: 10.1016/j.neuroscience.2017.09.035. 28951325

[84] Fan X, Wei W, Huang J, et al. Isoorientin attenuates cisplatin-induced nephrotoxicity through the inhibition of oxidative stress and apoptosis via activating the SIRT1/SIRT6/Nrf-2 pathway. Front Pharmacol. 2020;11:264. doi: 10.3389/fphar.2020.00264. 32256355 PMC7093647

[85] Barber MF, Michishita-Kioi E, Xi Y, et al. SIRT7 links H3K18 deacetylation to maintenance of oncogenic transformation. Nature. 2012;487(7405):114–118. doi: 10.1038/nature11043. 22722849 PMC3412143

[86] Sanchez-Navarro A, Martinez-Rojas MA, Albarran-Godinez A, et al. Sirtuin 7 deficiency reduces inflammation and tubular damage induced by an episode of acute kidney injury. Int J Mol Sci. 2022;23(5):2573. doi: 10.3390/ijms23052573. PMC8910458 35269715

[87] Chen G, Xue H, Zhang X, et al. p53 inhibition attenuates cisplatin-induced acute kidney injury through microRNA-142-5p regulating SIRT7/NF-kappaB. Ren Fail. 2022;44(1):368–380. doi: 10.1080/0886022X.2022.2039195. 35220863 PMC8890533

[88] Miyasato Y, Yoshizawa T, Sato Y, et al. Sirtuin 7 deficiency ameliorates cisplatin-induced acute kidney injury through regulation of the inflammatory response. Sci Rep. 2018;8(1):5927. doi: 10.1038/s41598-018-24257-7. 29651144 PMC5897539

[89] Kim MS, Blake M, Baek JH, et al. Inhibition of histone deacetylase increases cytotoxicity to anticancer drugs targeting DNA. Cancer Res. 2003;63(21):7291–7300. 14612526

[90] Wakamatsu T, Nakahashi Y, Hachimine D, et al. The combination of glycyrrhizin and lamivudine can ­reverse the cisplatin resistance in hepatocellular carcinoma cells through inhibition of multidrug resistance-associated proteins. Int J Oncol. 2007;31(6):1465–1472. doi: 10.3892/ijo.31.6.1465. 17982673

[91] Shin DH, Choi YJ, Park JW. SIRT1 and AMPK mediate hypoxia-induced resistance of non-small cell lung cancers to cisplatin and doxorubicin. Cancer Res. 2014;74(1):298–308. doi: 10.1158/0008-5472.CAN-13-2620. 24240701

[92] Asgar MA, Senawong G, Sripa B, et al. Synergistic anticancer effects of cisplatin and histone deacetylase inhibitors (SAHA and TSA) on cholangiocarcinoma cell lines. Int J Oncol. 2016;48(1):409–420. doi: 10.3892/ijo.2015.3240. 26575528

[93] Yang Z, Wang T, Wang F, et al. Discovery of selective histone deacetylase 6 inhibitors using the quinazoline as the cap for the treatment of cancer. J Med Chem. 2016;59(4):1455–1470. doi: 10.1021/acs.jmedchem.5b01342. 26443078

[94] Chen J-H, Zheng Y-L, Xu C-Q, et al. Valproic acid (VPA) enhances cisplatin sensitivity of non-small cell lung cancer cells via HDAC2 mediated down regulation of ABCA1. Biol Chem. 2017;398(7):785–792. doi: 10.1515/hsz-2016-0307. 28002023

[95] Higuchi T, Yamamoto J, Sugisawa N, et al. PPARγ agonist pioglitazone in combination with cisplatinum arrests a chemotherapy-resistant osteosarcoma PDOX model. Cancer Genomics Proteomics. 2020;17(1):35–40. doi: 10.21873/cgp.20165. 31882549 PMC6937125

[96] Liu T, Guan F, Wang Y, et al. MS-275 combined with cisplatin exerts synergistic antitumor effects in human esophageal squamous cell carcinoma cells. Toxicol Appl Pharmacol. 2020;395:114971. doi: 10.1016/j.taap.2020.114971. 32217144

[97] Mikami D, Kobayashi M, Uwada J, et al. β-Hydroxybutyrate enhances the cytotoxic effect of cisplatin via the inhibition of HDAC/survivin axis in human hepatocellular carcinoma cells. J Pharmacol Sci. 2020;142(1):1–8. doi: 10.1016/j.jphs.2019.10.007. 31757742

[98] Tavares MO, Milan TM, Bighetti-Trevisan RL, et al. Pharmacological inhibition of HDAC6 overcomes cisplatin chemoresistance by targeting cancer stem cells in oral squamous cell carcinoma. J Oral Pathol Med. 2022;51(6):529–537. doi: 10.1111/jop.13326. 35678235

[99] Mirzaei S, Gholami MH, Zabolian A, et al. Resveratrol augments doxorubicin and cisplatin chemotherapy: a novel therapeutic strategy. Curr Mol Pharmacol. 2023;16(3):280–306. doi: 10.2174/1874467215666220415131344. 35430977

[100] Jiang Q-Q, Fan L-y, Yang G-L, et al. Improved therapeutic effectiveness by combining liposomal honokiol with cisplatin in lung cancer model. BMC Cancer. 2008;8(1):242. doi: 10.1186/1471-2407-8-242. 18706101 PMC2543046

[101] Ramaiah MJ, Tangutur AD, Manyam RR. Epigenetic modulation and understanding of HDAC inhibitors in cancer therapy. Life Sci. 2021;277:119504. doi: 10.1016/j.lfs.2021.119504. 33872660

[102] Hyndman KA. Histone deacetylases in kidney physiology and acute kidney injury. Semin Nephrol. 2020;40(2):138–147. doi: 10.1016/j.semnephrol.2020.01.005. 32303277 PMC7172006

[103] Finnin MS, Donigian JR, Cohen A, et al. Structures of a histone deacetylase homologue bound to the TSA and SAHA inhibitors. Nature. 1999;401(6749):188–193. doi: 10.1038/43710. 10490031

[104] Ranganathan P, Hamad R, Mohamed R, et al. Histone deacetylase-mediated silencing of AMWAP expression contributes to cisplatin nephrotoxicity. Kidney Int. 2016;89(2):317–326. doi: 10.1038/ki.2015.326. 26509586 PMC4848209

[105] Liu J, Livingston MJ, Dong G, et al. Histone deacetylase inhibitors protect against cisplatin-induced acute kidney injury by activating autophagy in proximal tubular cells. Cell Death Dis. 2018;9(3):322. doi: 10.1038/s41419-018-0374-7. 29476062 PMC5833747

[106] Richard SA. Exploring the pivotal immunomodulatory and anti-inflammatory potentials of glycyrrhizic and glycyrrhetinic acids. Mediators Inflamm. 2021;2021:6699515–6699560. doi: 10.1155/2021/6699560. PMC7808814 33505216

[107] Ma T, Huang C, Meng X, et al. A potential adjuvant chemotherapeutics, 18β-glycyrrhetinic acid, inhibits renal tubular epithelial cells apoptosis via enhancing BMP-7 epigenetically through targeting HDAC2. Sci Rep. 2016;6(1):25396. doi: 10.1038/srep25396. 27145860 PMC4857087

[108] Shimazu T, Hirschey MD, Newman J, et al. Suppression of oxidative stress by beta-hydroxybutyrate, an endogenous histone deacetylase inhibitor. Science. 2013;339(6116):211–214. doi: 10.1126/science.1227166. 23223453 PMC3735349

[109] Milne JC, Lambert PD, Schenk S, et al. Small molecule activators of SIRT1 as therapeutics for the treatment of type 2 diabetes. Nature. 2007;450(7170):712–716. doi: 10.1038/nature06261. 18046409 PMC2753457

[110] Wang M-R, Zhang X-J, Liu H-C, et al. Matrine protects oligodendrocytes by inhibiting their apoptosis and enhancing mitochondrial autophagy. Brain Res Bull. 2019;153:30–38. doi: 10.1016/j.brainresbull.2019.08.006. 31404585

[111] Sears SM, Siskind LJ. Potential therapeutic targets for cisplatin-induced kidney injury: lessons from other models of AKI and fibrosis. J Am Soc Nephrol. 2021;32(7):1559–1567. doi: 10.1681/ASN.2020101455. 34049962 PMC8425641

[112] Gao Y, Dai X, Li Y, et al. Role of Parkin-mediated mitophagy in the protective effect of polydatin in sepsis-induced acute kidney injury. J Transl Med. 2020;18(1):114. doi: 10.1186/s12967-020-02283-2. 32131850 PMC7055075

[113] Li Z, Zhou L, Du Y, et al. Polydatin attenuates cisplatin-induced acute kidney injury via SIRT6-mediated autophagy activation. Oxid Med Cell Longev. 2022;2022:9035547. doi: 10.1155/2022/9035547. 36160707 PMC9507782

[114] Peng L, Liu D, Liu H, et al. Bombesin receptor-activated protein exacerbates cisplatin-induced AKI by regulating the degradation of SIRT2. Nephrol Dial Transplant. 2022;37(12):2366–2385. doi: 10.1093/ndt/gfac164. 35488871

[115] Huang X, Ma Y, Li Y, et al. Targeted drug delivery systems for kidney diseases. Front Bioeng Biotechnol. 2021;9:683247. doi: 10.3389/fbioe.2021.683247. 34124026 PMC8193852

[116] Feng L, Gu J, Yang Y, et al. Editorial: exploring the therapeutic effects of synthetic, semi-synthetic and naturally derived compounds against cancer. Front Pharmacol. 2023;14:1251835. doi: 10.3389/fphar.2023.1251835. 37675047 PMC10478079

[117] Radwan SM, Alqulaly M, Elsaeed MY, et al. L-carnitine reverses methotrexate-induced nephrotoxicity in experimental rat model: insight on SIRT1/PGC-1α/Nrf2/HO-1 axis. J Appl Toxicol. 2023;43(11):1667–1675. doi: 10.1002/jat.4503. 37312617

[118] Morsy MA, Ibrahim SA, Amin EF, et al. Curcumin ameliorates methotrexate-induced nephrotoxicity in rats. Adv Pharmacol Sci. 2013;2013:387071–387077. doi: 10.1155/2013/387071. 24381587 PMC3870078

[119] Uyumlu AB, Satılmış B, Atıcı B, et al. Phenethyl isothiocyanate protects against cyclophosphamide-induced nephrotoxicity via nuclear factor E2-related factor 2 pathway in rats. Exp Biol Med. 2023;248(2):157–164. doi: 10.1177/15353702221139206. PMC10041055 36598044

[120] Shati AA, El-Kott AF. Acylated ghrelin protects against doxorubicin-induced nephropathy by activating silent information regulator 1. Basic Clin Pharmacol Toxicol. 2021;128(6):805–821. doi: 10.1111/bcpt.13569. 33547742

